# Cancer Prevention for Survivors: Incidence of Second Primary Cancers and Sex Differences—A Population-Based Study from an Italian Cancer Registry

**DOI:** 10.3390/ijerph191912201

**Published:** 2022-09-26

**Authors:** Rosalia Ragusa, Antonina Torrisi, Alessia Anna Di Prima, Antonietta A. Torrisi, Antonella Ippolito, Margherita Ferrante, Anselmo Madeddu, Vincenzo Guardabasso

**Affiliations:** 1 HTA Committee, Azienda Ospedaliero Universitaria Policlinico “G. Rodolico—San Marco”, 95123 Catania, Italy; 2 Registro Tumori Integrato, Azienda Ospedaliero Universitaria Policlinico “G. Rodolico—San Marco”, 95123 Catania, Italy; 3 Department of Medical and Surgical Sciences and Advanced Technologies “G.F. Ingrassia”, University of Catania, 95123 Catania, Italy; 4 Registro Territoriale di Patologia Siracusa, Azienda Sanitaria Provinciale di Siracusa, 96100 Siracusa, Italy; 5 Research Promotion Office, Azienda Ospedaliero Universitaria Policlinico “G. Rodolico—San Marco”, 95123 Catania, Italy

**Keywords:** second primary cancer, multiple primary cancer, cancer registry, sex differences, cancer prevention, cancer survivors

## Abstract

Background: The number of cancer survivors continues to increase, thanks to advances in cancer diagnosis and treatment. Unfortunately, the incidence of a second primary cancer (SPC) is also increasing, but limited studies reporting incidence data are available regarding multiple cancers. This study presents our observations on multiple primary malignant cancers, the associations between sites, and the inherent sex differences. Patients and methods: We report the data, disaggregated by sex, concerning the SPCs that were recorded in the “Registro Tumori Integrato” (RTI) a population-based cancer registry in Sicily, Italy, as observed in the period from 2003 to 2017, in a total population of approximately 2,300,000. SPCs were divided into synchronous and metachronous cancers. *The International Classification of Diseases for Oncology*, third edition (ICD-O-3), was used for topographical and morphological classifications. Multiple primary cancers with multi-organ primitiveness were selected from the database of the RTI by extracting patients with more than one diagnosis. SPCs had different histology or morphology from the particular cancer that was considered to be the index cancer case. Multicenter or multifocal cancers, or metastases, were excluded. The percentages of cancer by sex and topography, the average age of incidence, and a breakdown by age were computed. Results: Differences were observed between sexes in terms of incidence and site for SPCs. The most frequent SPC was skin cancer (20% of the SPCs observed). The associations among sites of multiple cancers are reported. Conclusion: There are many gaps in our knowledge of sex differences in cancer. The study of multiple primary cancers could bring more likely opportunities for evaluation of the cancer burden and trends that can be used to identify new research areas by population health programs, as well as for clinical researchers.

## 1. Introduction

Improved life expectancy in the field of oncology involves an increase in second cancer risk after primary cancer treatment [1,2,3,4]. Given the successes that have been achieved in the survival of cancer patients, efforts regarding cancer prevention for survivors must now be increased. Nearly one in five of the cancers diagnosed today occurs in an individual with a previous diagnosis of cancer [5].

The increase in average life expectancy also indirectly involves an increase in the risk of the onset of cancer as it increases the time of exposure to carcinogens, but the reasons for the appearance of a second primary cancer (SPC) are not yet fully understood. Although risk factors often influence the development of a second cancer, we cannot predict whether or not a patient will develop a second cancer. 

An SPC may occur as a result of treatment for the first primary cancer (FPC) [6,7,8,9,10]. Specifically, some types of chemotherapy and radiation therapy [11,12] increase the risk of second cancer induction. However, an SPC may not necessarily be attributable to prior cancer treatment but may also reflect numerous combinations of the effects of host characteristics and environmental exposure [13]. Risk factors include the same elements that put people at risk for a first cancer, such as smoking and other tobacco use, being overweight, not getting regular physical activity, drinking too much alcohol, eating an unhealthy diet, and/or too much sun exposure.

Among the unmodifiable risk factors, we must keep in mind the factors of age and sex, as well as genetic predisposition. The phenomenon of SPCs has recently gained an important clinical relevance due to the large number of cancer patients who—having survived the first cancer—as they grow older, continue to be exposed to aging, the main risk factor for cancer [14,15,16,17,18]. 

Only recently have research studies been conducted to try to describe the drivers of differences based on sex seen in cancer patients [19]. Even less widespread is knowledge about sex-specific influences on the development of an SPC. 

Special attention should be given to multiple cancers because of the important impact on control strategies, in relation to the different risk factors and the different clinical frameworks in which they may arise. Patients with a prior cancer history are excluded from the majority of clinical trials; often, it is not easy to decide the treatment to be performed for the second cancer, given the rarity of the cases reported [20].

The objective of this work was to provide a descriptive analysis of SPCs in the four provinces covered by the “Registro Tumori Integrato” (RTI), the association between sites, and the differences between sexes, in order to evaluate the burden of second primary malignancies. 

## 2. Materials and Methods

### 2.1. Data Sources

The RTI has collected all incident cases of cancer since 2003 that were diagnosed in four topographical areas (the provinces of Catania (CT), Messina (ME), Syracuse (SR), and Enna (EN)), located in eastern Sicily, a large island in Southern Italy). The RTI is a population-based cancer registry that covers a total population of 2,264,298 (1,099,413 males, 1,164,885 females, as of 31 December 2017 (data from the National Statistical Institute (ISTAT), Rome, Italy). It was established in 2003 and is part of the network of Italian Cancer Registers (Associazione Italiana Registri Tumori, AIRTUM, Milan, Italy) [21]. Data in the RTI are deemed reliable, due to the use of international rules and classifications of the definition of the topography and morphology of cancer and for the assessment of the date of diagnosis and is subject to quality checks with CheckAIRTUM software (AIRTUM), total benchmark 93%; and with IARC-CHECK program (International Agency for Research on Cancer, IARC, Lyon, France) [22]. RTI participates in the collation of data for “Cancer Incidence in Five Continents” [23].

*The International Classification of Diseases for Oncology*, third edition (ICD-O-3), was used for creating topographical and morphological classifications, with some ad hoc groupings of ICD-O-3 codes for certain organ locations: e.g., colon–rectum, including C18–C21 (colon, recto-sigmoid junction, rectum, anus, and anal canal). The analyses included cases of melanoma and non-melanomatous skin cancers in C44. Cases based on the death certificate only, cases based on autopsy only, and cases with a follow-up time equal to zero were excluded from this analysis. To define the SPCs, we adopted rules from IARC—International Association of Cancer Registries (IACR) [24].

Patients newly diagnosed with cancer from 2003 to 2017 were followed over time from the first diagnosis of cancer until the date of death or the end of follow-up (31 December 2017), whichever came first. The date of death was derived from a standardized procedure of record linkage with the Regional Register of Death Causes, which is maintained by the Regional Government and provides death data for the whole regional population [25]. All cases were coded according to ICD-O-3. In the case of a second diagnosis of cancer, it was considered an SPC when it occurred within the period 2003–2017 and presented: (i) different topography and a different morphology or histotype; (ii) different topography and the same morphology or histotype; (iii) equal topography and a different morphology or histotype. 

Multicenter or multifocal cancers, or metastases, were excluded. 

The analyses included patients with FPC incidence diagnosed in 2003–2017 and excluded patients with a cancer incidence date prior to the beginning of RTI activity (2003). 

The percentages of cancers by sex and topography, the average age of incidence, and a breakdown by age (under 50 years old, between 50 and 69, and 70 or above) in the four provinces within the RTI geographical area were calculated.

For the present analysis, second primary cancers were defined as synchronous when there was a delay between the diagnosis of the FPC and the SPC of, at most, 6 months; otherwise, the second cancer was defined as metachronous [26]. It should be noted that different definitions, based on a 2-month cut-off time [27,28] or, more recently, a 4-month cut-off [29] have been proposed by other authors. In some synchronous cases where both diagnoses shared the same date, it was impossible to identify which cancer arose first; these cases were excluded from the attribution of the location of the first cancer and are included in tables within groups labeled “others”, as noted.

### 2.2. Statistical Considerations

Descriptive statistics were used in the tables as noted. Confidence intervals at the 95% probability level were computed for proportions, using the formula for normal approximation. The chi-squared test was used for comparisons of the rates, at the *p* = 0.05 level of false-positive probability.

The population averages for the calculation of raw incidence rates were obtained as averages of population, recorded at the beginning and on the last day of the year observed (that is, the first day of the following year), over the years under consideration. 

The available data for analyses concerning the incidence of SPCs were limited to patients having been diagnosed with an FPC in the years 2003–2007, in order to have a full 10-year span for follow-up for all patients. Therefore, the cohort of patients first diagnosed in 2003–2007 was extracted for these analyses.

### 2.3. Ethical Considerations

The present analysis was considered a secondary analysis of data already collected for the purposes of the RTI and, as such, it was covered by the same consent statements and privacy provisions of the RTI that had previously been obtained; therefore, a further request to the Ethics Committee was not deemed necessary under present national laws.

## 3. Results

### 3.1. Population Data and Incidence Rates 

The average total population covered by the RTI was 2,304,594 over the years 2003–2017. The province of Catania had 1,082,141 inhabitants, Messina had 649,024 inhabitants, Syracuse had 400,611 inhabitants, and Enna had 172,818 inhabitants. The total number of patients with malignant cancers that were diagnosed in the four provinces of the Registry was 165,179 (87,999 males and 77,180 females), with a raw incidence rate of 478/100,000 population (526 for males, 433 for females per 100,000 people). Detailed population data are reported in Table S1 of the Supplementary Materials, divided by province and sex. Detailed yearly population data are reported in Table S2 of the Supplementary Materials. Overall, the M/F ratio was 0.94 (range 0.92–0.97) in the years considered. 

In the RTI, we retrieved 176,527 new incident cases of cancers diagnosed between January 2003 and December 2017. In the last 15 years, the incidence of cancer has decreased in the male sex in all the provinces observed, while in women, the incidence has increased (Table S3 of the Supplementary Materials).

We identified 165,179 FPCs and 11,348 multiple primary cancers. These tumor cases occurred in 165,179 patients: of those, 154,647 patients showed only one incident of primary malignancy, and 10,532 patients had a history of multiple cancers (6.4% of cancer patients). Among those 10,532 patients who developed an SPC, 766 patients developed a third primary cancer, 48 patients developed a fourth primary cancer, and 2 patients developed a fifth primary cancer in their lifetime. 

The occurrence of further primary cancers after the second incident was not subjected to analyses. Among SPC patients, 64.2% were males, and the M/F ratio was 1.80.

Cancer incidence showed a lower incidence among women, both overall and in individual provinces, over the years considered (data in Table S3 of the Supplementary Materials). Although the sex ratio of M/F in the observed population was 0.94, indicating that females number more than males, the incidence of malignant neoplasms was higher in the male sex (M/F ratio 1.14). The incidence of cancer decreased in the male sex in all the observed provinces during the last 15 years. In women, the incidence increased.

### 3.2. Sites of Primary Cancers

Table 1 provides frequency data for FPCs and SPCs, according to sex and overall. The list of sites of diagnosed primary cancers registered in patients from the RTI, in order of frequency, is accumulated for all provinces encompassed by the RTI. The list of sites of FPCs observed, according to the site of the SPC, is also reported in Table 1 for all provinces. In order of frequency, the most frequent SPCs were skin cancers, followed by colon-rectum and lung cancers. When considered by province, the percentage of patients with an SPC ranged between 5.8% and 6.6% of those with cancer.

The most common pairs of FPCs and SPCs that were observed are reported in Table S4 of the Supplementary Materials. 

Overall, the site for the FPC most frequently associated with an SPC was the bladder, with 1680 cases (16.0% of all SPC). Other cancer sites showing a high prevalence of multiple cancers were, in order, prostate (14.9%), colon–rectum (13.8%), hematopoietic system (10.7%), and breast (10.6%). 

All other SPC sites did not reach 5% of the total SPCs. 

**Figure 1:**
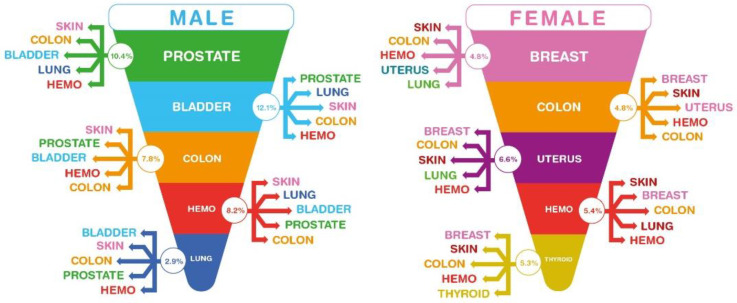
Second primary cancer sites, according to the site of the first primary cancer. FPCs are listed in white font and shown in decreasing order (the data can be found in Table S4 of the Supplementary Materials); percentages are shown of all SPCs, with the five most frequent sites listed in colored fonts, displayed in decreasing order from top to bottom (data in Table S5 of the Supplementary Materials).

Differences in incidence between sexes occurred for the FPC sites associated with SPCs, as shown in Figure 1. In male patients, the sites of FPCs that showed a high prevalence of SPCs were, in order: prostate, bladder, colon–rectum, hematopoietic system, and bronchus and lung; in the female sex, we observed cases of SPCs, especially in breast primary cancer, followed by cases of colon–rectum, uterus, hematopoietic system and thyroid gland cancers. 

**Table 1:** Sites of first primary cancers (FPCs) and of second primary cancers (SPCs) according to decreasing total occurrence.

First Primary Cancers (FPC)	ICD-O-3 Code	M		F		Total	
	Topography	*n*	(%)	*n*	(%)	*n*	(%)
Breast	C50	197	(0.2)	22,782	(29.5)	22,979	(13.9)
Colon, rectum, and anus	C18–C21	11,352	(12.9)	9668	(12.5)	21,020	(12.7)
Bronchus and lung	C34	13,012	(14.8)	3661	(4.7)	16,673	(10.1)
Hematopoietic system and lymph nodes	C42, C77	8842	(10.0)	7121	(9.2)	15,963	(9.7)
Prostate gland	C61	15,057	(17.1)			15,057	(9.1)
Bladder	C67	11,575	(13.2)	2350	(3.0)	13,925	(8.4)
Thyroid gland	C73	1704	(1.9)	5604	(7.3)	7308	(4.4)
Stomach	C16	3347	(3.8)	2383	(3.1)	5730	(3.5)
Uterus	C53–C55			5670	(7.4)	5670	(3.4)
Pancreas	C25	2445	(2.8)	2545	(3.3)	4990	(3.0)
Liver	C22	3243	(3.7)	1604	(2.1)	4847	(2.9)
Skin	C44	2215	(2.5)	1692	(2.2)	3907	(2.4)
Kidney	C64	2116	(2.4)	1147	(1.5)	3263	(2.0)
Gallbladder	C23	1207	(1.4)	1511	(2.0)	2718	(1.6)
Ovary	C56			2465	(3.2)	2465	(1.5)
Larynx	C32	1723	(2.0)	171	(0.2)	1894	(1.1)
Esophagus	C15	384	(0.4)	145	(0.2)	529	(0.3)
Duodenum	C17	277	(0.3)	229	(0.3)	506	(0.3)
Other *		9303	(10.6)	6432	(8.3)	15,735	(9.5)
**Total**		**87,999**	**(100.0)**	**77,180**	**(100.0)**	**165,179**	**(100.0)**
**Second Primary Cancers**	**ICD-O-3 code**	**M**		**F**		**Total**	
(SPC)	Topography	*n*	(%)	*n*	(%)	*n*	(%)
Skin	C44	1420	(21.0)	642	(17.0)	2062	(19.6)
Colon, rectum, anus	C18–C21	785	(11.6)	477	(12.7)	1262	(12.0)
Bronchus and lung	C34	912	(13.5)	230	(6.1)	1142	(10.8)
Prostate gland	C61	878	(13.0)			878	(8.3)
Bladder	C67	726	(10.7)	140	(3.7)	866	(8.2)
Hematopoietic system and lymph nodes	C42, C77	472	(7.0)	310	(8.2)	782	(7.4)
Breast	C50	14	(0.2)	578	(15.3)	592	(5.6)
Stomach	C16	216	(3.2)	117	(3.1)	333	(3.2)
Pancreas	C25	178	(2.6)	130	(3.5)	308	(2.9)
Kidney	C64	209	(3.1)	92	(2.4)	301	(2.9)
Thyroid gland	C73	76	(1.1)	185	(4.9)	261	(2.5)
Uterus	C53–C55			246	(6.5)	246	(2.3)
Liver	C22	170	(2.5)	62	(1.6)	232	(2.2)
Ovary	C56			193	(5.1)	193	(1.8)
Gallbladder	C23	89	(1.3)	59	(1.6)	148	(1.4)
Larynx	C32	89	(1.3)	12	(0.3)	101	(1.0)
Duodenum	C17	27	(0.4)	21	(0.6)	48	(0.5)
Esophagus	C15	36	(0.5)	6	(0.2)	42	(0.4)
Other **		468	(6.9)	267	(7.1)	735	(6.9)
**Total**		**6765**	**(100.0)**	**3767**	**(100.0)**	**10,532**	**(100.0)**

Notes: ICD-O-3: *International Classification of Diseases for Oncology*, third edition; * “Other” includes cancer cases from all other ICD-O-3 codes; ** “Other” cancer sites include other ICD-O-3 codes and 523 cases with synchronous cancers where the first cancer could not be ascertained.

### 3.3. Sites of Second Primary Cancers

Overall, the most frequent site for an SPC was skin cancer (C44), with 2062 cases, of which 18.1% were associated with prostate cancer. Skin cancer was followed in frequency by an association with hematologic neoplasms, colon–rectum, bladder, breast cancers, primary skin cancers, and, more rarely, associations with other sites. 

**Figure 2:**
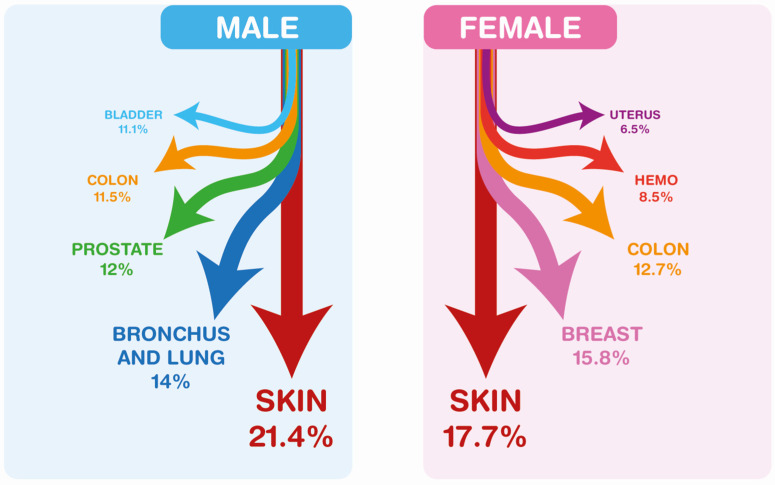
Sites of second primary cancers, shown by decreasing frequency of occurrence. The most frequent SPC sites are detailed, in decreasing order from bottom to top; other cancers, combined, represented 30.0% in males and 38.8% in females (data in Table S5 of the Supplementary Materials).

Table S5 of the Supplementary Materials details the various sites of SPCs associated with the most common FPCs. The sites of SPCs were different between males and females. Figure 2 shows the most frequent sites of SPCs according to the decreasing frequency of occurrence in both sexes. In both sexes, skin remained the most frequent site of second cancers. In the male patients, we then found, in decreasing order, bronchus and lung, prostate, colon–rectum, and bladder cancers. In the female patients, after skin, the most frequent SPCs were of the breast, colon–rectum, hematopoietic system, and uterus.

There were 523 subjects with a simultaneous diagnosis (same date) of two different primary cancers. These cases were excluded from the attribution of the location of the first cancer and are included in Tables S4 and S5 of the Supplementary Materials, in the group labeled “others”.

### 3.4. Incidence of First Primary Cancers and Second Primary Cancers, Stratified by Age Class

**Figure 3:**
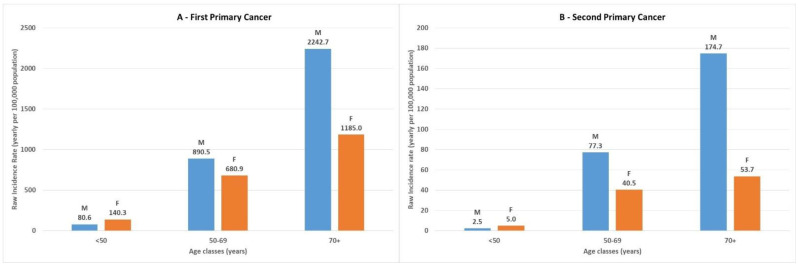
The raw incidence rate data in patients from RTI 2003–2017, by age class, at diagnosis of the FPC. M, males, F, females; <50 = aged 0–49; 50–69 = aged 50 to 69 years old; 70+ = aged 70 and above.

Figure 3 reports the raw incidence rate of primary cancer when stratified by sex and age classes. A higher occurrence of cancer among women in people under 50 years of age (M/F ratio 0.57) can be observed both in FPCs and in SPCs (M/F ratio 0.50). 

In the 50–69 age group, men had a higher occurrence of cancers than women, both in primary cancers (M/F 1.31) and in SPCs (M/F 1.91). 

The occurrence of multiple cancers was much higher (M/F 3.25) in men than in women of older age (70+), compared to the occurrence of primary cancers (M/F 1.89), despite the fact that women in this population live longer.

### 3.5. Incidence of Second Primary Cancers in the Cohort Subgroup of 2003–2007

Table 2 reports the percent frequency of SPCs in the cohort of FPCs diagnosed in 2003–2007 and observed until 2017. The analysis was limited to this subgroup of available data, in order to have a complete minimum 10-year follow-up for all patients. The percentage frequencies, in reference to the date of onset of the second cancer, are reported overall and after dividing the time course into 3 periods: synchronous cases, onset after 6 months and within 5 years (60 months), and onset after 5 years. In this cohort, SPCs were observed in 11.1% of male patients with FPCs and in 7.1% of female patients. In the table, the frequencies of SPC diagnoses for the 5 most frequent sites of primary cancer are reported in detail.

**Table 2:** Percentage frequency of a second primary cancer (SPC) in the years 2003–2017, in cancer patients diagnosed in 2003–2007 with a first primary cancer (FPC), in overall data, and within 6 months (synchronous) or metachronous: between 7 months and 5 years (7–60 months) from the first diagnosis, or after 5 years (>60 months).

Patients with First Primary Cancer (FPC)		2003–2007	FPC Patients with Occurrence of SPCs in 2003–2017				Synchronous		Metachronous			
							0–6 mo.		7–60 mo.		>60 Months	
**All cancers**		n	n	%	(95%	CI)	n	%	n	%	n	%
	M	29,164	3243	11.1	(10.8	−11.5)	500	15.4	1348	41.6	1395	43.0
	F	24,376	1730	7.1	(6.8	−7.4)	211	12.2	718	41.5	801	46.3
												
**Breast**												
	F	6772	502	7.4	(6.8	−8.0)	31	6.2	216	43.0	255	50.8
												
**Colon-Rectum**												
	M	3647	454	12.4	(11.4	−13.5)	72	15.8	195	43.0	187	41.2
	F	3098	247	8.0	(7.0	−8.9)	40	16.2	107	43.3	100	40.5
												
**Lung**												
	M	4535	175	3.9	(3.3	−4.4)	46	26.3	73	41.7	56	32.0
	F	995	23	2.3	(1.4	−3.2)	7	30.5	9	39.1	7	30.4
												
**Prostate**												
	M	4904	759	15.5	(14.5	−16.5)	99	13.0	321	42.3	339	44.7
												
**Bladder**												
	M	3961	728	18.4	(17.2	−19.6)	121	16.6	301	41.3	306	42.1
	F	756	84	11.1	(8.9	−13.4)	15	17.9	30	35.7	39	46.4

Note: M males; F females; n = counts; % = percent frequency; 95% CI = 95% confidence interval.

The time elapsed between the first diagnosis of the primary cancer and the diagnosis of an SPC can be studied as the event-free time interval, representing the delay time for the onset of the SPC. The average delay for a metachronous case, excluding synchronous cases within 6 months, was 68.6 months (median 55 months). The most frequent time delay in metachronous SPCs was 8 months.

**Figure 4:**
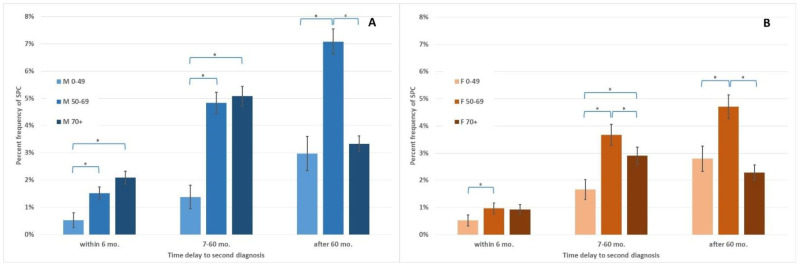
Percentage incidence of a second primary cancer in patients, 2003–2007. **Panel A**: M, males; **Panel B**: F, females. Age ranges at the diagnosis of an FPC: 0–49 years old; 50–69 years old; 70+ = age 70 and above. Second diagnosis was within a time frame of 6 months (synchronous), between 7 and 60 months, or after 60 months (five years) during follow-up. Note: * significantly different, *p* < 0.05, chi-squared tests; error bars: 95% confidence intervals.

The percentage frequency of SPCs varied with age in both sexes (Figure 4) in the 2003–2007 cohort studied. 

The percentage frequency of SPCs was computed for all SPCs after the diagnosis of FPCs (overall), or separately for three follow-up ranges: within 6 months, after 6 months and within 5 years (7–60 months), and after 5 years of follow-up (after 60 months).

The incidence of SPCs overall and of the 5 most frequent cancers in the population studied (breast in females, colon–rectum, lung, prostate, and bladder), divided by age group, is reported in Table S6 of the Supplementary Materials. 

## 4. Discussion

Therapeutic efforts in oncology, in the third millennium, should also consider survival after an SPC. Survivors of SPCs are now a growing reality; however, guidelines for the tertiary prevention of cancer are still limited. Currently, most protocols only provide for a follow-up of the FPC to detect the early signs of possible recurrence or the appearance of secondary metastases.

The purpose of this study was to explore epidemiological views regarding SPCs, using data from a fairly large population cancer registry, the RTI in Eastern Sicily, Italy, which encompasses four provinces with a total population of about 2,300,000. The therapeutic successes obtained in the last 30 years in oncology treatment, the identification of lifestyle risk factors for developing cancer, and advances in the identification of genetic associations as predisposing factors will need to be evaluated for SPC detection and treatment. Future projects could evaluate whether SPC frequencies are decreasing over time or are remaining stable and whether the time passing until an SPC is increasing as well. This would provide a population-level evaluation of treatment effectiveness for a broad range of FPCs. 

The reasons for the appearance of an SPC are not yet fully understood, and it is important to underscore the relevance of SPC data analysis for cancer survivors. Risk model studies were conducted for predicting the risk of an SPC and for profiling the clinical characteristic of cancer survivors who remain at high risk of developing SPCs, but this topic needs further exploration [30,31,32,33]. 

Data collected from population-based cancer registries of SPCs can help to distinguish, within the field of multiple cancers, the most frequent occurrences from the rarest associations. This could help in understanding the biological mechanisms underlying these associations and the neoplastic late effects of treatment and could suggest possible implications in terms of treatment. In fact, if the possibility of a second tumor in a certain location is proven to be likely, the treatment of the first one could be constructed with a different strategy, or, at least, a different follow-up strategy could be implemented [20]. Unlike studies that report the incidence of multiple primary malignant neoplasms from a single institution, the population registry data, as in the RTI to which we had access, are certainly more complete and methodologically more reliable for a correct portrayal of this issue. In fact, those studies deriving from the collection of data from a single institution, although interesting, are often affected by the selective characteristics of the institution and do not include those who, for various reasons, were not assisted by the same institution [34,35].

An understanding of the associations between FPCs and SPCs could be useful to identify possible factors for the success of SPC treatment and to improve prevention (if linked to lifestyles or iatrogenic treatments) or the early detection of SPCs (in the case of unchangeable factors such as gender or genetic age). 

### 4.1. Sites of FPCs

The incidence of cancer decreased in the male sex in all the observed provinces during the last 15 years, while, in women, the incidence increased, as a likely result of breast and Pap test screening campaigns [31,36,37,38]. FPCs that are most frequently associated with an SPC are different in the two sexes. In men, cancer of the prostate is the most frequent and this is also the site that, with greater frequency, has been associated with a second tumor. Cancer of the bladder, which in our work was found to be the most frequent site associated with SPCs among all patients, is associated in men with a second tumor in the prostate, then with bronchus and lung cancers. As has been proposed in the literature, the finding of a high frequency of prostate cancer diagnosis in patients with bladder cancer may be due to the random finding of prostate cancers during a cystectomy performed for bladder carcinoma [39].

Colorectal cancer (CRC) survivors of both sexes remain at high risk of developing an SPC, so much so that some authors have developed a tool to predict the risk of a second cancer, based on individual factors [30]. We found an SPC in 6.9% of all patients with CRC. Skin, bladder, and prostate cancers were the most frequent SPCs in male colon cancer survivors [40], whereas breast cancer was the most frequent second cancer in females with an initial colon cancer diagnosis, suggesting that the surveillance of prostate cancer in men and breast cancer in women should include cancer screening or active surveillance protocols in follow-up management. In our study, we also observed associations between CRC and hemopoietic cancer or another colon subsite in both sexes. We also observed associations between CRC and uterus cancer in women, as previously reported in the literature [41,42]. Rarely have we found an association between CRC and second primary pancreatic cancer in either sex, as has been reported by others [43].

In primary lung cancer, although this is frequent as a site of the primary tumor, an SPC occurred in our data only rarely (4% of total primary lung cancer), which is probably due to a worse prognosis.

In women, breast cancer was the most frequent FPC, and an SPC occurred in 4.8% of breast FPCs. However, due to the high frequency of breast cancer in women, SPCs occurring after a breast FPC made up 30.4% of all SPCs in women.

The most frequent SPC that we observed after a primary breast cancer in females were cancers of the skin, colon–rectum, and the hematopoietic system. Some authors noted that breast cancer is associated with a significant increase in chronic myeloid leukemia risk [44,45]; other authors reported frequent breast SPC, followed by cancers of the bronchus and lung, uterus, thyroid, and melanoma [46]. 

Corso et al. [47] reported the occurrence of SPCs, such as digestive tract cancer, melanoma, uterus, ovarian, and thyroid cancers after breast FPCs. 

Besides some classification differences, our results were similar, but showed a higher number of SPCs in the bronchus and lung than in the ovaries or thyroid; this might be related to different smoking habits, but no data are available for assessing possible exposures. Clinical surveillance also appears necessary to prevent CRCs, uterus, ovarian, and thyroid cancers in women with an FPC of the breast.

We found that the other FPCs associated with an SPC in females were, in order of frequency, colon–rectum, uterus, hematopoietic system, and thyroid.

### 4.2. Sites of SPC

When considering the location site of SPCs, it is pivotal to consider separately the SPCs observed by sex, to be able to derive indications useful in clinical oncology practice.

It is relevant to note that the most frequent SPCs in both sexes was skin cancer (Table S5 of the Supplementary Materials), which also represented 20% of the SPCs observed (21% male and 17% female). Our analyses included cases of melanoma and non-melanomatous skin cancers in C44; some cancer registries do not collect non-melanoma skin cancers and might report lower frequencies. At any rate, the importance of surveillance of skin moles or suspected skin lesions in cancer patients should not be underestimated. 

Among the other frequent sites of SCP, we found in the male sex that bronchus and lung, prostate, colon-rectum, and bladder were the most frequent; in the female sex, the breast was found to be the most frequent site of SCP, followed by, in order of frequency, colon–rectum, blood and lymphatic system, and uterus cancers.

It should be noted that among the 523 cases with a simultaneous diagnosis that were excluded from the assessment of the primary site because it is not distinguishable, we have frequently found in men an association between prostate and bladder cancer, and, in women, the association between cancers of the uterus and ovary.

According to age, the percentage of SPCs reached the highest frequency in the age groups of 50–69 years, but some differences in the incidence of SPCs between the two sexes were noted.

Secondary cancers are more frequent in women under 50 years of age. In men, the incidence increases in the following age groups much more frequently than in women, reaching threefold that of women who are over 70.

The time of onset of SPCs is different in different FPC sites: in breast cancer, an SPC occurred after 5 years in more than 50% of cases; in other locations, most SPCs occurred within 5 years (Table 2).

These data underline the importance of timely screening in women under 50 years of age, who, when suffering from breast cancer, should continue to have regular, appropriate follow-ups, even after 5 years from the first diagnosis, and should have an individualized breast cancer screening protocol, especially if they are younger than the screening program age eligibility requirement.

An SPC is a rare occurrence. The associations reported in the literature mostly ascribe the appearance of SPCs to iatrogenic factors but, certainly, the genetic or biological factors of the individual could promote or protect from SPC, being equal to the treatment carried out [48,49,50,51,52]. Lifestyle choices, such as increased physical activity or tobacco cessation, might reduce some SPCs. Moreover, the possible occurrence of SPCs should always be considered in directing clinicians to use the minimal effective treatment of an FPC, saving further, more aggressive treatments for the successful treatment of a possible SPC.

These observations on SPCs should be considered for personalized cancer follow-up recommendations, including tailored screening for selected sites that are more frequently associated with the FPC, and for longer than 5 years post-diagnosis of the FPC.

We plan to investigate the relationships between the different histological types and the sites of SPCs. The histological report and the type of treatment performed may provide more information concerning the need for more extended monitoring of the FPC and the associations with an SPC. It will be useful to verify whether the onset of a second cancer will remain low in subjects with a longer duration of follow-up, at 15 or 20 years after the first diagnosis.

If, as proposed, genetic factors are the main cause of SPC, these must be researched, considering the differences between men and women. The occurrence of cancer is less frequent in women, who have a longer average lifespan, suggesting that genetic mechanisms for repair after carcinogenicity might be more efficient compared to men; the study of the molecular mechanisms underlying sex differences in cancer will be a step forward in an era of personalized medicine [53].

Finally, further studies are needed concerning the difference between the incidence of SPCs in the two biological sexes with the incidence of SPCs in the various genders, to verify whether lifestyle, habits, drugs and treatments of various kinds may change the percentages or associations described.

### 4.3. Limitations

The RTI is a population-based cancer registry, wherein detailed data on treatments and treatment protocols, their changes over time, and the influence of screening policies are not available and their influences, therefore, cannot be appreciated.

Data on the patients’ possible exposures to carcinogens and their smoking habits were not available for causal hypotheses.

## 5. Conclusions

There are many sex differences in cancer, from epidemiology to etiology and from management to outcomes. There are sex-related differences in response to medical therapy, radiation therapy, and surgery. Many factors, most of which are not yet well understood, are at the basis of the phenomena that lead to the development, possibly after a long time, of a second tumor in another location than the primary cancer location.

Given the different associations among the topographic sites of FPCs and SPCs between sexes, it will be necessary to provide separate paths for men/women in terms of the follow-up of primary cancers. A follow-up of the FPC should also include, in addition to diagnostic investigations for possible relapses or metastases of the FPC, the development of a cancer screening and surveillance plan for the most frequent locations of possible SPCs. 

In view of the number of cases of SPCs observed in the skin in both sexes, it is advisable to recommend a skin examination in all patients. Cancer follow-ups could be extended beyond 5 years since primary tumors can be followed by an SPC after a substantial amount of time.

SPCs are expected to increase, due to the longer survival of cancer patients with a treatable FPC. Differences between sexes were found in the various SPC sites and in the different associations between FPC and SPC locations. Future research that incorporates clinical and patient data can provide insights into the etiology of SPCs, as well as into their specific locations. In addition, looking into clinical and behavioral interventions that can reduce the risk of a second cancer could narrow the sex gap in terms of the second cancer risk. Monitoring cancer registry data for trends in the detection of SPCs and their locations for different age groups and genders can also lead to new research directions regarding cancer screening and prevention programs.

## Data Availability

The data that support the findings of our study are available on request from the Integrated Cancer Registry (RTI CT-ME-EN). Further information is available from the corresponding author upon request.

## Supplementary Materials

The following supporting information can be downloaded at: https://www.mdpi.com/article/10.3390/ijerph191912201/s1. Table S1: Summary of data for years 2003–2017; Table S2: Population in the four provinces of the RTI; Table S3: Cancer incidence rates (per 100,000 population) by province, sex, and year of diagnosis, age-adjusted to the 2013 European Standard Population; Table S4: Observed cases of second primary cancers according to the site of first primary cancer; Table S5: Observed cases of first primary cancers, according to the site of second primary cancer; Table S6: Cancer cases (2003–2007) and the percentage frequency of second primary cancers (2003–2017), by sex and age.

## Abbreviations

AIRTUM	Associazione Italiana Registri Tumori (Italian Cancer Registries Association)
CRC	Colorectal cancer
CT	Province of Catania (Sicily, Italy)
EN	Province of Enna (Sicily, Italy)
FPC	First primary cancer
IACR	International Association of Cancer Registries
IARC	International Agency for Research on Cancer
ICD-O-3	*International Classification of Diseases for Oncology*, third edition
ME	Province of Messina (Sicily, Italy)
RTI	Registro Tumori Integrato (Integrated Cancer Registry)
SEER	Surveillance, epidemiology and end results
SPC	Second primary cancer(s)
SR	Province of Syracuse (Sicily, Italy)
