# Supplementary material for: Cancer Prevention for Survivors: Incidence of Second Primary Cancers and Sex Differences—A Population-Based Study from an Italian Cancer Registry
Source: Int J Environ Res Public Health. 2022 Sep 26;19(19):12201. doi: 10.3390/ijerph191912201 (PMC9565941; doi:10.3390/ijerph191912201)
Supplement: Supplementary file 1 [file ijerph-19-12201-s001.zip › ijerph-1883195-supplementary.pdf]

Supplementary Materials

Table S1: Summary of data for years 2003-2017

		Population (average)			Cancer patients			Raw incidence rates*		
Province		M	F	Total	M	F	Total	M	F	Total
Catania	CT	523,428	558,713	1,082,141	40,211	36,396	76,607	512	434	472
Enna	EN	83,216	89,602	172,818	6,299	5,246	11,545	505	390	445
Messina	ME	312,190	336,834	649,024	26,197	23,047	49,244	559	456	506
Syracuse	SR	196,699	203,912	400,611	15,292	12,491	27,783	518	408	462
Total		1,115,532	1,189,062	2,304,594	87,999	77,180	165,179	526	433	478

Notes:

Average population 2003-2017 (Data from https://demo.istat.it)

Cancer data from RTI consolidated Cancer Registry for the provinces of CT Catania, EN Enna, ME Messina, SR Syracuse, in Eastern Sicily, Italy; number of cancer patients over years 2003-2017

* Raw incidence rates per 100,000 population/year

Table S2: Population in the four Provinces of the RTI

Province	Sex	2003	2004	2005	2006	2007	2008	2009	2010	2011	2012	2013	2014	2015	2016	2017	2018*
CT	M	510,026	513,545	515,617	518,111	519,231	521,826	523,934	525,971	528,201	529,006	529,487	529,516	529,636	528,882	527,070	524,781
CT	F	546,179	548,776	551,012	553,694	554,809	558,211	560,505	562,546	564,336	565,223	565,522	565,149	564,275	562,218	559,911	557,042
CT	Total	1,056,205	1,062,321	1,066,629	1,071,805	1,074,040	1,080,037	1,084,439	1,088,517	1,092,537	1,094,229	1,095,009	1,094,665	1,093,911	1,091,100	1,086,981	1,081,823
EN	M	84,760	84,431	84,294	84,238	84,069	84,290	84,261	84,205	84,014	83,896	83,296	82,636	81,955	81,296	80,309	79,502
EN	F	91,674	91,301	91,001	90,989	90,780	90,962	91,020	90,871	90,747	90,355	89,503	88,844	87,967	86,977	85,987	84,657
EN	Total	176,434	175,732	175,295	175,227	174,849	175,252	175,281	175,076	174,761	174,251	172,799	171,480	169,922	168,273	166,296	164,159
ME	M	316,404	316,589	315,988	315,086	314,407	314,404	314,846	314,731	315,206	314,295	312,494	310,791	308,710	306,240	303,895	300,952
ME	F	342,437	342,164	341,671	340,568	339,705	340,121	340,630	340,198	339,881	338,748	336,728	334,808	332,533	329,370	326,594	323,195
ME	Total	658,841	658,753	657,659	655,654	654,112	654,525	655,476	654,929	655,087	653,043	649,222	645,599	641,243	635,610	630,489	624,147
SR	M	193,834	194,217	194,662	194,862	195,280	196,913	198,329	198,662	199,209	199,485	198,912	198,237	197,799	196,850	195,748	194,178
SR	F	202,052	202,467	202,928	203,221	203,443	204,546	205,477	206,064	206,758	206,655	205,715	204,874	204,009	202,767	201,629	199,991
SR	Total	395,886	396,684	397,590	398,083	398,723	401,459	403,806	404,726	405,967	406,140	404,627	403,111	401,808	399,617	397,377	394,169
Total	M	1,105,024	1,108,782	1,110,561	1,112,297	1,112,987	1,117,433	1,121,370	1,123,569	1,126,630	1,126,682	1,124,189	1,121,180	1,118,100	1,113,268	1,107,022	1,099,413
Total	F	1,182,342	1,184,708	1,186,612	1,188,472	1,188,737	1,193,840	1,197,632	1,199,679	1,201,722	1,200,981	1,197,468	1,193,675	1,188,784	1,181,332	1,174,121	1,164,885
Grand	Total	2,287,366	2,293,490	2,297,173	2,300,769	2,301,724	2,311,273	2,319,002	2,323,248	2,328,352	2,327,663	2,321,657	2,314,855	2,306,884	2,294,600	2,281,143	2,264,298

Notes:

Label	Province	Area
CT	Catania	3,553 Km^2
EN	Enna	2,574 Km^2
ME	Messina	3,266 Km^2
SR	Siracusa	2,124 Km^2

M males; F females

Population upon January 1 of each year; * population on Jan. 1, 2018 conventionally considered equal to population on Dec. 31, preceding year.

Data from ISTAT, Italian Statistical Institute, Rome, Italy. from https://demo.istat.it "Ricostruzione intercensuaria della popolazione, Italiani + stranieri residenti" retrieved 26/3/2021

Table S3 Cancer Incidence Rates (per 100,000 population) by Province, sex and year of diagnosis, age-adjusted to the 2013 European Standard Population

Province	Sex	2003	2004	2005	2006	2007	2008	2009	2010	2011	2012	2013	2014	2015	2016	2017
CT	M	481.9	466.4	437.8	437.5	446.4	437.2	426.5	424.4	438.5	434.6	412.6	415.8	403.0	405.5	396.2
CT	F	337.1	336.7	333.3	330.9	328.3	354.1	340.2	340.8	346.7	343.9	346.3	318.1	332.6	351.4	331.4
EN	M	318.3	372.4	335.6	414.2	450.7	438.5	383.3	385.8	426.7	378.6	364.8	347.8	335.9	363.7	313.5
EN	F	219.0	279.6	236.6	296.7	269.2	291.7	290.0	299.3	322.3	293.4	268.9	282.1	269.0	306.0	285.7
ME	M	426.6	416.1	410.1	410.0	410.7	416.6	422.6	399.4	385.6	417.6	421.2	417.7	411.0	400.9	373.1
ME	F	296.1	297.6	315.9	311.4	324.4	329.9	326.0	318.6	325.5	331.9	313.2	325.4	320.5	332.5	318.3
SR	M	401.0	441.4	411.1	425.6	393.8	395.5	404.4	406.5	433.4	417.8	394.0	404.3	402.5	369.9	370.0
SR	F	284.3	294.3	296.2	293.8	281.4	307.0	312.2	290.7	334.6	299.5	315.1	327.5	316.7	305.3	293.7
Total	M	436.6	438.3	415.8	424.4	426.7	423.2	418.0	411.1	420.7	422.2	408.1	409.6	400.8	395.7	381.8
	F	305.6	312.9	313.9	315.8	313.9	333.7	327.5	321.9	336.4	328.8	326.0	319.2	322.1	335.3	319.6

Notes:

Province labels: CT Catania; EN Enna; ME Messina; SR Siracusa.

M males; F females

Table S4: Observed cases of second primary cancers according to the site of first primary cancer.

First Primary Cancer site (ICD-O-3 topography code)	Second Primary Cancer (SPC) site	ICD-O-3 topography code	M		F		Total		% of First Cancer Site §		
			n	(%)	n	(%)	n	(%) of all SPC	overall (%)	in M (%)	in F (%)
Bladder (C67)	Prostate Gland	C61	353	(5.5)			353	(3.5)	(2.5)	(3.0)	
	Bronchus and Lung	C34	310	(4.8)	15	(0.4)	325	(3.2)	(2.3)	(2.7)	(0.6)
	Skin	C44	219	(3.4)	23	(0.7)	242	(2.4)	(1.7)	(1.9)	(1.0)
	Colon, rectum	C18-C21	133	(2.1)	26	(0.7)	159	(1.6)	(1.1)	(1.1)	(1.1)
	Hematopoietic system and lymph nodes	C42, C77	86	(1.3)	19	(0.5)	105	(1.0)	(0.8)	(0.7)	(0.8)
	Liver	C22	44	(0.7)	6	(0.2)	50	(0.5)	(0.4)	(0.4)	(0.3)
	Stomach	C16	33	(0.5)	7	(0.2)	40	(0.4)	(0.3)	(0.3)	(0.3)
	Breast	C50	1	(0.0)	39	(1.1)	40	(0.4)	(0.3)	(0.0)	(1.7)
	Pancreas	C25	35	(0.5)	4	(0.1)	39	(0.4)	(0.3)	(0.3)	(0.2)
	Kidney	C64	28	(0.4)	3	(0.1)	31	(0.3)	(0.2)	(0.2)	(0.1)
	Bladder	C67	9	(0.1)	0	(0.0)	9	(0.1)	(0.1)	(0.1)	(0.0)
	Thyroid gland	C73	5	(0.1)	3	(0.1)	8	(0.1)	(0.1)	(0.0)	(0.1)
	Uterus	C53-C55			5	(0.1)	5	(0.1)	(0.0)		(0.2)
	*Other**		*141*	*(2.2)*	*20*	*(0.6)*	*161*	*(1.6)*	*(1.2)*	*(1.2)*	*(0.9)*
**Total**			**1,397**	**(21.7)**	**170**	**(4.8)**	**1,567**	**(16.0)**	**(11.3)**	**(12.1)**	**(7.2)**
Prostate Gland (C61)	Skin	C44	373	(5.8)			373	(3.7)	(2.5)	(2.5)	
	Colon, rectum	C18-C21	270	(4.2)			270	(2.7)	(1.8)	(1.8)	
	Bladder	C67	247	(3.8)			247	(2.5)	(1.6)	(1.6)	
	Bronchus and Lung	C34	182	(2.8)			182	(1.8)	(1.2)	(1.2)	
	Hematopoietic system and lymph nodes	C42, C77	134	(2.1)			134	(1.3)	(0.9)	(0.9)	
	Stomach	C16	67	(1.0)			67	(0.7)	(0.4)	(0.4)	
	Kidney	C64	51	(0.8)			51	(0.5)	(0.3)	(0.3)	
	Liver	C22	35	(0.5)			35	(0.3)	(0.2)	(0.2)	
	Pancreas	C25	35	(0.5)			35	(0.3)	(0.2)	(0.2)	
	Thyroid gland	C73	11	(0.2)			11	(0.1)	(0.1)	(0.1)	
	Breast	C50	7	(0.1)			7	(0.1)	(0.0)	(0.0)	
	*Other**		*152*	*(2.4)*			*152*	*(1.5)*	*(1.0)*	*(1.0)*	
**Total**			**1,564**	**(24.2)**			**1,564**	**(16.0)**	**(10.4)**	**(10.4)**	
Colon, rectum (C18-C21)	Skin	C44	172	(2.7)	75	(2.1)	247	(2.5)	(1.2)	(1.5)	(0.8)
	Bladder	C67	123	(1.9)	20	(0.6)	143	(1.4)	(0.9)	(1.1)	(0.2)
	Prostate Gland	C61	135	(2.1)			135	(1.3)	(0.9)	(1.2)	
	Bronchus and Lung	C34	106	(1.6)	23	(0.6)	129	(1.3)	(0.9)	(0.9)	(0.2)
	Hematopoietic system and lymph nodes	C42, C77	73	(1.1)	48	(1.4)	121	(1.2)	(0.8)	(0.6)	(0.5)
	Breast	C50	2	(0.0)	97	(2.7)	99	(1.0)	(0.7)	(0.0)	(1.0)
	Colon, rectum	C18-C21	50	(0.8)	30	(0.8)	80	(0.8)	(0.5)	(0.4)	(0.3)

First Primary Cancer site (ICD-O-3 topography code)	Second Primary Cancer (SPC) site	ICD-O-3 topography code	M n	M (%)	F n	F (%)	Total n	Total (%) of all SPC	% of First Cancer Site § overall (%)	in M (%)	in F (%)
	Stomach	C16	35	(0.5)	19	(0.5)	54	(0.5)	(0.4)	(0.3)	(0.2)
	Uterus	C53-C55			50	(1.4)	50	(0.5)	(0.3)		(0.5)
	Pancreas	C25	26	(0.4)	14	(0.4)	40	(0.4)	(0.3)	(0.2)	(0.1)
	Liver	C22	27	(0.4)	12	(0.3)	39	(0.4)	(0.3)	(0.2)	(0.1)
	Kidney	C64	27	(0.4)	11	(0.3)	38	(0.4)	(0.3)	(0.2)	(0.1)
	Thyroid gland	C73	12	(0.2)	9	(0.3)	21	(0.2)	(0.1)	(0.1)	(0.1)
	*Other**		*97*	*(1.5)*	*60*	*(1.7)*	*157*	*(1.6)*	*(1.0)*	*(0.9)*	*(0.6)*
**Total**			**885**	**(13.7)**	**468**	**(13.2)**	**1,353**	**(14.0)**	**(9.0)**	**(7.8)**	**(4.8)**
Hematopoietic system and lymph nodes (C42, C77)	Skin	C44	205	(3.2)	91	(2.6)	296	(3.0)	(1.4)	(1.8)	(0.9)
	Colon, rectum	C18-C21	72	(1.1)	47	(1.3)	119	(1.2)	(0.6)	(0.6)	(0.5)
	Bronchus and Lung	C34	91	(1.4)	22	(0.6)	113	(1.1)	(0.5)	(0.8)	(0.2)
	Bladder	C67	86	(1.3)	13	(0.4)	99	(1.0)	(0.5)	(0.8)	(0.1)
	Prostate Gland	C61	75	(1.2)			75	(0.7)	(0.4)	(0.7)	
	Breast	C50	1	(0.0)	52	(1.5)	53	(0.5)	(0.3)	(0.0)	(0.5)
	Stomach	C16	28	(0.4)	15	(0.4)	43	(0.4)	(0.2)	(0.2)	(0.2)
	Hematopoietic system and lymph nodes	C42, C77	25	(0.4)	14	(0.4)	39	(0.4)	(0.2)	(0.2)	(0.1)
	Liver	C22	25	(0.4)	9	(0.3)	34	(0.3)	(0.2)	(0.2)	(0.1)
	Pancreas	C25	21	(0.3)	13	(0.4)	34	(0.3)	(0.2)	(0.2)	(0.1)
	Kidney	C64	23	(0.4)	10	(0.3)	33	(0.3)	(0.2)	(0.2)	(0.1)
	Uterus	C53-C55			26	(0.7)	26	(0.3)	(0.1)		(0.3)
	Thyroid gland	C73	11	(0.2)	11	(0.3)	22	(0.2)	(0.1)	(0.1)	(0.1)
	*Other**		*63*	*(1.0)*	*57*	*(1.6)*	*120*	*(1.2)*	*(0.6)*	*(0.6)*	*(0.6)*
**Total**			**726**	**(11.3)**	**380**	**(10.7)**	**1,106**	**(11.0)**	**(5.3)**	**(6.4)**	**(3.9)**
Breast (C50)	Skin	C44	6	(0.1)	213	(6.0)	219	(2.2)	(1.0)	(3.0)	(0.9)
	Colon, rectum	C18-C21	0	(0.0)	163	(4.3)	163	(1.6)	(0.7)	(0.0)	(0.7)
	Hematopoietic system and lymph nodes	C42, C77	1	(0.0)	111	(3.1)	112	(1.1)	(0.5)	(0.5)	(0.5)
	Uterus	C53-C55			91	(2.6)	91	(0.9)	(0.4)		(0.4)
	Bronchus and lung	C34	2	(0.0)	80	(2.2)	82	(0.8)	(0.4)	(1.0)	(0.4)
	Thyroid gland	C73	0	(0.0)	75	(2.1)	75	(0.7)	(0.3)	(0.0)	(0.3)
	Bladder	C67	3	(0.0)	56	(1.6)	59	(0.6)	(0.3)	(1.5)	(0.2)
	Stomach	C16	0	(0.0)	44	(1.2)	44	(0.4)	(0.2)	(0.0)	(0.2)
	Pancreas	C25	1	(0.0)	42	(1.2)	43	(0.4)	(0.2)	(0.5)	(0.2)
	Kidney	C64	1	(0.0)	26	(0.7)	27	(0.3)	(0.1)	(0.5)	(0.1)
	Liver	C22	0	(0.0)	17	(0.5)	17	(0.2)	(0.1)	(0.0)	(0.1)
	Breast	C50	0	(0.0)	9	(0.3)	9	(0.1)	(0.0)	(0.0)	(0.0)
	Prostate Gland	C61	5	(0.1)			5	(0.0)	(0.0)	(2.5)	
	*Other**		*3*	*(0.0)*	*156*	*(4.4)*	*159*	*(1.6)*	*(0.7)*	*(1.5)*	*(0.7)*
**Total**			**22**	**(0.3)**	**1,083**	**(30.4)**	**1,105**	**(11.0)**	**(4.8)**	**(11.2)**	**(4.8)**
Bronchus and lung (C34)	Bladder	C67	89	(1.4)	4	(0.1)	93	(0.9)	(0.6)	(0.7)	(0.1)
	Skin	C44	64	(1.0)	10	(0.3)	74	(0.7)	(0.4)	(0.5)	(0.3)

First Primary Cancer site (ICD-O-3 topography code)	Second Primary Cancer (SPC) site	ICD-O-3 topography code	M		F		Total		% of First Cancer Site §		
			n	(%)	n	(%)	n	(%) of all SPC	overall (%)	in M (%)	in F (%)
	Colon, rectum	C18-C21	57	(0.9)	9	(0.3)	66	(0.7)	(0.4)	(0.4)	(0.2)
	Prostate Gland	C61	35	(0.5)			35	(0.3)	(0.2)	(0.3)	
	Hematopoietic system and lymph nodes	C42, C77	26	(0.4)	7	(0.2)	33	(0.3)	(0.2)	(0.2)	(0.2)
	Pancreas	C25	18	(0.3)	6	(0.2)	24	(0.2)	(0.1)	(0.1)	(0.2)
	Stomach	C16	14	(0.2)	1	(0.0)	15	(0.1)	(0.1)	(0.1)	(0.0)
	Breast	C50	0	(0.0)	11	(0.3)	11	(0.1)	(0.1)	(0.0)	(0.3)
	Kidney	C64	8	(0.1)	0	(0.0)	8	(0.1)	(0.0)	(0.1)	(0.0)
	Liver	C22	4	(0.1)	2	(0.1)	6	(0.1)	(0.0)	(0.0)	(0.1)
	Thyroid gland	C73	4	(0.1)	2	(0.1)	6	(0.1)	(0.1)	(0.0)	(0.1)
	Bronchus and Lung	C34	5	(0.1)	0	(0.0)	5	(0.0)	(0.0)	(0.0)	(0.0)
	Uterus	C53-C55			2	(0.1)	2	(0.0)	(0.0)		(0.1)
	*Other**		*50*	*(0.8)*	*15*	*(0.4)*	*65*	*(0.6)*	*(0.4)*	*(0.4)*	*(0.4)*
Total			**374**	**(5.8)**	**69**	**(1.9)**	**443**	**(4.0)**	**(2.7)**	**(2.9)**	**(1.9)**
Thyroid gland C73	Breast	C50	0	(0.0)	103	(2.9)	103	(1.0)	(1.4)	(0.0)	(1.8)
	Skin	C44	24	(0.4)	45	(1.3)	69	(0.7)	(0.9)	(1.4)	(0.8)
	Colon, rectum	C18-C21	10	(0.2)	31	(0.9)	41	(0.4)	(0.6)	(0.6)	(0.6)
	Hematopoietic system and lymph nodes	C42, C77	15	(0.2)	25	(0.7)	40	(0.4)	(0.5)	(0.9)	(0.4)
	Bronchus and Lung	C34	13	(0.2)	19	(0.5)	32	(0.3)	(0.4)	(0.8)	(0.3)
	Uterus	C53-C55			17	(0.5)	17	(0.2)	(0.2)		(0.3)
	Bladder	C67	8	(0.1)	9	(0.3)	17	(0.2)	(0.2)	(0.5)	(0.2)
	Prostate Gland	C61	16	(0.2)			16	(0.2)	(0.2)	(0.9)	
	Kidney	C64	6	(0.1)	9	(0.3)	15	(0.1)	(0.2)	(0.4)	(0.2)
	Pancreas	C25	2	(0.0)	4	(0.1)	6	(0.1)	(0.1)	(0.1)	(0.1)
	Stomach	C16	1	(0.0)	3	(0.1)	4	(0.0)	(0.1)	(0.1)	(0.1)
	Thyroid gland	C73	0	(0.0)	2	(0.1)	2	(0.0)	(0.0)	(0.0)	(0.0)
	Liver	C22	0	(0.0)	1	(0.0)	1	(0.0)	(0.0)	(0.0)	(0.0)
	*Other**		*22*	*(0.3)*	*34*	*(1.0)*	*56*	*(0.6)*	*(0.8)*	*(1.3)*	*(0.6)*
Total			**117**	**(1.8)**	**302**	**(8.5)**	**419**	**(4.0)**	**(5.7)**	**(6.9)**	**(5.4)**
Uterus C53-C55	Breast	C50			96	(2.7)	96	(1.0)	(1.7)		(1.7)
	Colon, rectum	C18-C21			42	(1.2)	42	(0.4)	(0.7)		(0.7)
	Skin	C44			39	(1.1)	39	(0.4)	(0.7)		(0.7)
	Bronchus and Lung	C34			32	(0.9)	32	(0.3)	(0.6)		(0.6)
	Hematopoietic system and lymph nodes	C42, C77			23	(0.7)	23	(0.2)	(0.4)		(0.4)
	Bladder	C67			18	(0.5)	18	(0.2)	(0.2)		(0.3)
	Pancreas	C25			16	(0.4)	16	(0.2)	(0.3)		(0.3)
	Thyroid gland	C73			15	(0.4)	15	(0.1)	(0.3)		(0.3)
	Stomach	C16			9	(0.3)	9	(0.1)	(0.2)		(0.2)
	Kidney	C64			9	(0.3)	9	(0.1)	(0.2)		(0.2)
	Uterus	C53-C55			8	(0.2)	8	(0.1)	(0.1)		(0.1)

First Primary Cancer site (ICD-O-3 topography code)	Second Primary Cancer (SPC) site	ICD-O-3 topography code	M n	M (%)	F n	F (%)	Total n	Total (%) of all SPC	% of First Cancer Site § overall (%)	in M (%)	in F (%)
	Liver	C22			3	(0.1)	3	(0.0)	(0.1)		(0.1)
	*Other**				*65*	*(1.8)*	*65*	*(0.6)*	*(1.1)*		*(1.1)*
Total					**375**	**(10.5)**	**375**	**(4.0)**	**(6.6)**		**(6.6)**
Skin	C44		158	(2.4)	111	(3.1)	269	(3.0)	(6.9)	(7.1)	(6.6)
Kidney	C64		183	(2.8)	71	(2.0)	254	(3.0)	(7.8)	(8.6)	(6.2)
Stomach	C16		162	(2.5)	82	(2.3)	244	(2.0)	(4.3)	(4.8)	(3.4)
Liver	C22		107	(1.7)	39	(1.1)	146	(1.0)	(3.0)	(3.3)	(2.4)
Pancreas	C25		32	(0.5)	19	(0.5)	51	(1.0)	(1.0)	(1.3)	(0.7)
*Other**			*723*	*(11.2)*	*390*	*(11.0)*	*1,113*	*(11.0)*	*(5.3)*	*(6.4)*	*(4.0)*
**Grand Total**			**6,450**	**(100.0)**	**3,559**	**(100.0)**	**10,009**	**(100.0)**	**(6.1)**	**(7.3)**	**(4.6)**

Notes: ICD-O-3 International Classification of Diseases for Oncology - version 3; * *Other* [cancer sites] includes other ICD-O-3 codes; M males; F females; § number of first primary cancer by site provided in Table 1 of manuscript.

Table S5: Observed cases of first primary cancers according to the site of second primary cancer

Second Primary Cancer (SPC) site (ICD-O-3 topography code)	Site of First Primary Cancer	ICD-O-3 topography code	M n	M (%)	F n	F (%)	Total n	Total (%) of all SPC	% of First Cancer Site § overall (%)	in M (%)	in F (%)
Skin C44	Prostate Gland	C61	373	(5.8)			373	(3.7)	(18.5)	(27.0)	
	Hematopoietic system and lymph nodes	C42, C77	205	(3.2)	91	(2.6)	296	(3.0)	(14.7)	(14.8)	(14.5)
	Colon, rectum	C18-C21	172	(2.7)	75	(2.1)	247	(2.5)	(12.3)	(12.4)	(11,9)
	Bladder	C67	219	(3.4)	23	(0.7)	242	(2.4)	(12.0)	(15.8)	(3.7)
	Breast	C50	6	(0.1)	213	(6.0)	219	(2.2)	(10.9)	(0.4)	(33.9)
	Skin	C44	61	(0.9)	37	(1.0)	98	(1.0)	(4.9)	(4.4)	(5.9)
	Bronchus and lung	C34	64	(1.0)	10	(0.3)	74	(0.7)	(3.7)	(4.6)	(1.6)
	Thyroid gland	C73	24	(0.4)	45	(1.3)	69	(0.7)	(3.4)	(1.7)	(7.2)
	Stomach	C16	37	(0.6)	9	(0.3)	46	(0.5)	(2.3)	(2.7)	(1.4)
	Kidney	C64	31	(0.5)	12	(0.3)	43	(0.4)	(2.1)	(2.2)	(1.9)
	Liver	C22	34	(0.5)	8	(0.2)	42	(0.4)	(2.1)	(2.5)	(1.3)
	Uterus	C53-C55			39	(1.1)	39	(0.4)	(1.9)		(6.2)
	Pancreas	C25	2	(0.0)	3	(0.1)	5	(0.0)	(0.2)	(0.1)	(0.5)
	*Other**		*155*	*(2.4)*	*64*	*(1.8)*	*219*	*(2.2)*	*(10.9)*	*(11.2)*	*(10.2)*
**Total**			**1,383**	**(21.4)**	**629**	**(17.7)**	**2,012**	**(20.0)**	**(100)**	**(100.0)**	**(100.0)**
Colon-rectum (C18-21)	Prostate Gland	C61	270	(4.0)			270	(2.7)	(22.6)	(36.4)	
	Breast	C50	0	(0.0)	163	(4.6)	163	(1.6)	(13.6)	(0.0)	(36.0)
	Bladder	C67	133	(2.1)	26	(0.7)	159	(1.6)	(13.3)	(17.9)	(5.7)
	Hematopoietic system and lymph nodes	C42, C77	72	(1.1)	47	(1.3)	119	(1.2)	(10.0)	(9.7)	(10.4)
	Colon-rectum	C18-C21	50	(0.8)	30	(0.8)	80	(0.8)	(6.7)	(6.7)	(6.6)
	Bronchus and lung	C34	57	(0.9)	9	(0.3)	66	(0.7)	(5.5)	(7.7)	(2.0)
	Uterus	C53-C55			42	(1.2)	42	(0.4)	(3.5)		(9.3)
	Thyroid gland	C73	10	(0.2)	31	(0.9)	41	(0.4)	(3.4)	(1.3)	(6.8)
	Kidney	C64	26	(0.4)	12	(0.3)	38	(0.4)	(3.2)	(3.5)	(2.6)
	Stomach	C16	22	(0.3)	14	(0.4)	36	(0.4)	(3.0)	(3.0)	(3.1)
	Skin	C44	19	(0.3)	10	(0.3)	29	(0.3)	(2.4)	(2.6)	(2.2)
	Liver	C22	11	(0.2)	3	(0.1)	14	(0.1)	(1.2)	(1.5)	(0.7)
	Pancreas	C25	6	(0.1)	3	(0.1)	9	(0.1)	(0.8)	(0.8)	(0.7)
	*Other**		*66*	*(1.0)*	*63*	*(1.8)*	*129*	*(1.3)*	*(10.8)*	*(8.9)*	*(13.9)*
**Total**			**742**	**(11.5)**	**453**	**(12.7)**	**1,195**	**(12.0)**	**(100)**	***(100.0)***	***(100.0)***
Bronchus and lung (C34)	Bladder	C67	310	(4.8)	15	(0.4)	325	(3.2)	(28.7)	(34.4)	(6.6)
	Prostate Gland	C61	182	(2.8)			182	(1.8)	(16.1)	(20.2)	
	Colon-rectum	C18-C21	106	(1.6)	23	(0.6)	129	(1.3)	(11.4)	(11.8)	(10.0)
	Hematopoietic system and lymph nodes	C42, C77	91	(1.4)	22	(0.6)	113	(1.1)	(10.0)	(10.1)	(9.6)

Second Primary Cancer (SPC) site (ICD-O-3 topography code)	Site of First Primary Cancer	ICD-O-3 topography code	M		F		Total		% of First Cancer Site §		
			n	(%)	n	(%)	n	(%) of all SPC	overall (%)	in M (%)	in F (%)
	Breast	C50	2	(0.0)	80	(2.2)	82	(0.8)	(7.3)	(0.2)	(34.9)
	Uterus	C53-C55			32	(0.9)	32	(0.3)	(2.8)		(14.0)
	Thyroid gland	C73	13	(0.2)	19	(0.5)	32	(0.3)	(2.8)	(1.4)	(8.3)
	Kidney	C64	21	(0.3)	4	(0.1)	25	(0.2)	(2.0)	(2.3)	(1.7)
	Stomach	C16	18	(0.3)	1	(0.0)	19	(0.2)	(1.7)	(2.0)	(0.4)
	Skin	C44	13	(0.2)	6	(0.2)	19	(0.2)	(1.7)	(1.4)	(2.6)
	Liver	C22	14	(0.2)	1	(0.0)	15	(0.1)	(1.3)	(1.6)	(0.4)
	Pancreas	C25	4	(0.1)	1	(0.0)	5	(0.0)	(0.4)	(0.4)	(0.4)
	Bronchus and lung	C34	5	(0.1)	0	(0.0)	5	(0.0)	(0.4)	(0.6)	(0.0)
	*Other**		*123*	*(1.9)*	*25*	*(0.7)*	*148*	*(1.5)*	*(13.1)*	*(13.6)*	*(10.9)*
**Total**			**902**	**(14.0)**	**229**	**(6.4)**	**1,131**	**(11.0)**	**(100)**	**(100.0)**	**(100.0)**
Bladder (C67)	Prostate Gland	C61	247	(3.8)			247	(2.5)	(28.9)	(34.5)	
	Colon-rectum	C18-C21	123	(1.9)	20	(0.6)	143	(1.4)	(16.7)	(17.2)	(14.5)
	Hematopoietic system and lymph nodes	C42, C77	86	(1.3)	13	(0.4)	99	(1.0)	(11.6)	(12.0)	(9.4)
	Bronchus and lung	C34	89	(1.4)	4	(0.1)	93	(0.9)	(10.9)	(12.4)	(2.9)
	Breast	C50	3	(0.0)	56	(1.6)	59	(0.6)	(6.9)	(0.4)	(40.6)
	Kidney	C64	23	(0.4)	2	(0.1)	25	(0.2)	(2.9)	(3.2)	(1.4)
	Stomach	C16	17	(0.3)	1	(0.0)	18	(0.2)	(2.1)	(2.4)	(0.7)
	Uterus	C53-C55			18	(0.5)	18	(0.2)	(2.1)		(13.0)
	Thyroid gland	C73	8	(0.1)	9	(0.3)	17	(0.2)	(2.0)	(1.1)	(6.5)
	Skin	C44	11	(0.2)	4	(0.1)	15	(0.1)	(1.8)	(1.5)	(2.9)
	Liver	C22	13	(0.2)	1	(0.0)	14	(0.1)	(1.6)	(1.8)	(0.7)
	Bladder	C67	9	(0.1)	0	(0.0)	9	(0.1)	(1.1)	(1.3)	(0.0)
	Pancreas	C25	4	(0.1)	1	(0.0)	5	(0.0)	(0.6)	(0.6)	(0.7)
	*Other**		*83*	*(1.3)*	*9*	*(0.3)*	*92*	*(0.9)*	*(10.8)*	*(11.6)*	*(6.5)*
**Total**			**716**	**(11.1)**	**138**	**(3.9)**	**854**	**(9.0)**	**(100)**	**(100.0)**	**(100.0)**
Prostate Gland (C61)	Bladder	C67	353	(5.5)			353	(3.5)	(45.7)	(45.7)	
	Colon-rectum	C18-C21	135	(2.1)			135	(1.3)	(17.5)	(17.5)	
	Hematopoietic system and lymph nodes	C42, C77	75	(1.2)			75	(0.7)	(9.7)	(9.7)	
	Bronchus and lung	C34	35	(0.5)			35	(0.3)	(4.5)	(4.5)	
	Kidney	C64	34	(0.5)			34	(0.3)	(4.4)	(4.4)	
	Stomach	C16	23	(0.4)			23	(0.2)	(3.0)	(3.0)	
	Thyroid gland	C73	16	(0.2)			16	(0.2)	(2.1)	(2.1)	
	Skin	C44	9	(0.1)			9	(0.1)	(1.2)	(1.2)	
	Breast	C50	5	(0.1)			5	(0.0)	(0.6)	(0.6)	
	Liver	C22	5	(0.1)			5	(0.0)	(0.6)	(0.6)	
	Pancreas	C25	4	(0.1)			4	(0.0)	(0.5)	(0.5)	
	*Other**		*78*	*(1.2)*			*78*	*(0.8)*	*(10.1)*	*(10.1)*	
**Total**			**772**	**(12.0)**			**772**	**(8.0)**	**(100)**	**(100.0)**	

Second Primary Cancer (SPC) site (ICD-O-3 topography code)	Site of First Primary Cancer	ICD-O-3 topography code	M		F		Total		% of First Cancer Site §		
			n	(%)	n	(%)	n	(%) of all SPC	overall (%)	in M (%)	in F (%)
Hematopoietic system and lymph nodes (C42, C77)	Prostate gland	C61	134	(2.1)			134	(1.3)	(17.8)	(29.6)	
	Colon-rectum	C18-C21	73	(1.1)	48	(1.4)	121	(1.2)	(16.1)	(16.2)	(15.9)
	Breast	C50	1	(0.0)	111	(3.1)	112	(1.1)	(14.9)	(0.2)	(36.9)
	Bladder	C67	86	(1.3)	19	(0.5)	105	(1.0)	(13.9)	(19.0)	(6.3)
	Thyroid gland	C73	15	(0.2)	25	(0.7)	40	(0.4)	(5.3)	(3.3)	(8.3)
	Hematopoietic system and lymph nodes	C42, C77	25	(0.4)	14	(0.4)	39	(0.4)	(5.2)	(5.5)	(4.7)
	Bronchus and lung	C34	26	(0.4)	7	(0.2)	33	(0.3)	(4.4)	(5.8)	(2.3)
	Uterus	C53-C55			23	(0.7)	23	(0.2)	(3.1)		(7.6)
	Stomach	C16	12	(0.2)	8	(0.2)	20	(0.2)	(2.7)	(2.7)	(2.7)
	Kidney	C64	12	(0.2)	8	(0.2)	20	(0.2)	(2.6)	(2.7)	(2.0)
	Liver	C22	10	(0.2)	7	(0.2)	17	(0.2)	(2.3)	(2.2)	(2.3)
	Skin	C44	7	(0.1)	3	(0.1)	10	(0.1)	(1.3)	(1.5)	(1.0)
	Pancreas	C25	5	(0.1)	1	(0.0)	6	(0.1)	(0.8)	(1.1)	(0.3)
	*Other**		*46*	*(0.7)*	*29*	*(0.8)*	*75*	*(0.7)*	*(10.0)*	*(10.2)*	*(9.6)*
**Total**			**452**	**(7.0)**	**301**	**(8.5)**	**753**	**(8.0)**	**(100)**	***(100.0)***	***(100.0)***
Breast (C50)	Thyroid gland	C73	0	(0.0)	103	(2.9)	103	(1.0)	(17.9)	(0.0)	(18.3)
	Colon-rectum	C18-C21	2	(0.0)	97	(2.7)	99	(1.0)	(17.2)	(14.3)	(17.3)
	Uterus	C53-C55			96	(2.7)	96	(1.0)	(16.7)		(17.1)
	Hematopoietic system and lymph nodes	C42, C77	1	(0.0)	52	(1.5)	53	(0.6)	(9.2)	(7.1)	(9.3)
	Bladder	C67	1	(0.0)	39	(1.1)	40	(0.4)	(6.9)	(7.1)	(6.9)
	Skin	C44	1	(0.0)	22	(0.6)	23	(0.2)	(4.0)	(7.1)	(3.9)
	Stomach	C16	1	(0.0)	21	(0.6)	22	(0.2)	(3.8)	(7.1)	(3.7)
	Kidney	C64	0	(0.0)	17	(0.5)	17	(0.2)	(3.0)	(0.0)	(3.0)
	Liver	C22	1	(0.0)	15	(0.4)	16	(0.2)	(2.8)	(7.1)	(2.7)
	Bronchus and lung	C34	0	(0.0)	11	(0.3)	11	(0.1)	(1.9)	(0.0)	(2.0)
	Breast	C50	0	(0.0)	9	(0.3)	9	(0.1)	(1.6)	(0.0)	(1.6)
	Prostate gland	C61	7	(0.1)			7	(0.1)	(1.2)	(50.0)	
	Pancreas	C25	0	(0.0)	5	(0.1)	5	(0.0)	(0.9)	(0.0)	(0.9)
	*Other**		*0*	*(0.0)*	*75*	*(2.1)*	*75*	*(0.7)*	*(13.0)*	*(0.0)*	*(13.3)*
**Total**			**14**	**(0.2)**	**562**	**(15.8)**	**576**	**(6.0)**	**(100)**	**(100.0)**	**(100.0)**
Stomach C16	Prostate gland	C61	67	(1.0)			67	(0.7)	(20.6)	(31.9)	
	Colon-rectum	C18-C21	35	(0.5)	19	(0.5)	54	(0.5)	(16.6)	(16.7)	(16.5)
	Breast	C50	0	(0.0)	44	(1.2)	44	(0.4)	(13.5)	(0.0)	(38.3)
	Hematopoietic system and lymph nodes	C42, C77	28	(0.4)	15	(0.4)	43	(0.4)	(13.2)	(13.3)	(13.0)
	Bladder	C67	33	(0.5)	7	(0.2)	40	(0.4)	(12.3)	(15.7)	(6.1)
	Bronchus and lung	C34	14	(0.2)	1	(0.0)	15	(0.1)	(4.6)	(6.7)	(0.9)

Second Primary Cancer (SPC) site (ICD-O-3 topography code)	Site of First Primary Cancer	ICD-O-3 topography code	M n	M (%)	F n	F (%)	Total n	Total (%) of all SPC	% of First Cancer Site § overall (%)	% of First Cancer Site § in M (%)	% of First Cancer Site § in F (%)
	Uterus	C53-C55			9	(0.3)	9	(0.1)	(2.8)		(7.8)
	Kidney	C64	6	(0.1)	1	(0.0)	7	(0.1)	(2.2)	(2.9)	(0.9)
	Skin	C44	3	(0.0)	1	(0.0)	4	(0.0)	(1.2)	(1.4)	(0.9)
	Thyroid gland	C73	1	(0.0)	3	(0.1)	4	(0.0)	(1.2)	(0.5)	(2.6)
	Stomach	C16	1	(0.0)	1	(0.0)	2	(0.0)	(0.6)	(0.5)	(0.9)
	Pancreas	C25	1	(0.0)	1	(0.0)	2	(0.0)	(0.6)	(0.5)	(0.9)
	Liver	C22	0	(0.0)	1	(0.0)	1	(0.0)	(0.3)	(0.0)	(0.9)
	*Other**		*21*	*(0.3)*	*12*	*(0.3)*	*33*	*(0.3)*	*(10.2)*	*(10.0)*	*(10.4)*
**Total**			**210**	**(3.3)**	**115**	**(3.2)**	**325**	**(3.0)**	**(100)**	**(100.0)**	**(100.0)**
Pancreas C25	Breast	C50	1	(0.0)	42	(1.2)	43	(0.4)	(15.0)	(0.6)	(34.4)
	Colon-rectum	C18-C21	26	(0.4)	14	(0.4)	40	(0.4)	(13.9)	(15.8)	(11.5)
	Bladder	C67	35	(0.5)	4	(0.1)	39	(0.4)	(13.6)	(21.2)	(3.3)
	Prostate gland	C61	35	(0.5)			35	(0.3)	(12.2)	(21.2)	
	Hematopoietic system and lymph nodes	C42, C77	21	(0.3)	13	(0.4)	34	(0.3)	(11.8)	(12.7)	(10.7)
	Bronchus and lung	C34	18	(0.3)	6	(0.2)	24	(0.2)	(8.4)	(10.9)	(4.9)
	Uterus	C53-C55			16	(0.4)	16	(0.2)	(5.6)		(13.1)
	Skin	C44	5	(0.1)	3	(0.1)	8	(0.1)	(2.8)	(3.0)	(2.5)
	Stomach	C16	2	(0.0)	6	(0.2)	8	(0.1)	(2.8)	(1.2)	(4.9)
	Thyroid gland	C73	2	(0.0)	4	(0.1)	6	(0.1)	(2.1)	(1.2)	(3.3)
	Kidney	C64	3	(0.0)	2	(0.1)	5	(0.0)	(1.7)	(1.8)	(1.6)
	Liver	C22	3	(0.0)	0	(0.0)	3	(0.1)	(1.0)	(1.8)	(0.0)
	*Other**		*14*	*(0.2)*	*12*	*(0.3)*	*26*	*(0.3)*	*(9.1)*	*(8.5)*	*(9.8)*
**Total**			**165**	**(2.6)**	**122**	**(3.4)**	**287**	**(3.0)**	**(100)**	**(100.0)**	**(100.0)**
Kidney C64	Prostate gland	C61	51	(0.8)			51	(0.5)	(19.4)	(28.2)	
	Colon-rectum	C18-C21	27	(0.4)	11	(0.3)	38	(0.4)	(14.4)	(14.9)	(13.4)
	Hematopoietic system and lymph nodes	C42, C77	23	(0.4)	10	(0.3)	33	(0.3)	(12.5)	(12.7)	(12.2)
	Bladder	C67	28	(0.4)	3	(0.1)	31	(0.3)	(11.8)	(15.5)	(3.7)
	Breast	C50	1	(0.0)	26	(0.7)	27	(0.3)	(10.3)	(0.6)	(31.7)
	Thyroid gland	C73	6	(0.1)	9	(0.3)	15	(0.1)	(5.7)	(3.3)	(11.0)
	Skin	C44	10	(0.2)	4	(0.1)	14	(0.1)	(5.3)	(5.5)	(4.9)
	Uterus	C34			9	(0.3)	9	(0.1)	(3.4)		(11.0)
	Stomach	C16	6	(0.1)	2	(0.1)	8	(0.1)	(3.0)	(3.3)	(2.4)
	Bronchus and lung	C34	8	(0.1)	0	(0.0)	8	(0.1)	(3.0)	(4.4)	(0.0)
	Pancreas	C25	3	(0.0)	2	(0.1)	5	(0.0)	(1.9)	(1.7)	(2.4)
	Liver	C22	2	(0.0)	0	(0.0)	2	(0.0)	(0.8)	(1.1)	(0.0)
	*Other**		*16*	*(0.2)*	*6*	*(0.2)*	*22*	*(0.2)*	*(8.4)*	*(8.8)*	*(7.3)*
**Total**			**181**	**(2.8)**	**82**	**(2.3)**	**263**	**(3.0)**	**(100)**	**(100.0)**	**(100.0)**
Uterus	Breast	C50			91	(2.6)	13	(0.9)	(39.4)		(39.4)

Second Primary Cancer (SPC) site (ICD-O-3 topography code)	Site of First Primary Cancer	ICD-O-3 topography code	M		F		Total		% of First Cancer Site §		
			n	(%)	n	(%)	n	(%) of all SPC	overall (%)	in M (%)	in F (%)
C53-C55	Colon-rectum	C18-C21			50	(1.4)	50	(0.5)	(21.6)		(21.6)
	Hematopoietic system and lymph nodes	C42, C77			26	(0.7)	26	(0.3)	(11.3)		(11.3)
	Thyroid gland	C73			17	(0.5)	17	(0.2)	(7.4)		(7.4)
	Uterus	C53-C55			8	(0.2)	8	(0.1)	(3.5)		(3.5)
	Skin	C44			7	(0.2)	7	(0.1)	(3.0)		(3.0)
	Bladder	C67			5	(0.1)	5	(0.0)	(2.2)		(2.2)
	Stomach	C16			3	(0.1)	3	(0.0)	(1.3)		(1.3)
	Bronchus and lung	C34			2	(0.1)	2	(0.0)	(0.9)		(0.9)
	Kidney	C64			2	(0.1)	2	(0.0)	(0.9)		(0.9)
	*Other**				*20*	*(0.6)*	*20*	*(0.2)*	*(8.7)*		*(8.7)*
**Total**					**231**	**(6.5)**	**231**	**(2.0)**	**(100)**		**(100.0)**
Thyroid gland C73	Breast	C50	0	(0.0)	75	(2.1)	75	(0.7)	(32.8)	(0.0)	(47.2)
	Hematopoietic system and lymph nodes	C42, C77	11	(0.2)	11	(0.3)	23	(0.2)	(9.6)	(15.7)	(6.9)
	Colon-rectum	C18-C21	12	(0.2)	9	(0.3)	21	(0.2)	(9.2)	(17.1)	(5.7)
	Uterus	C53-C55			15	(0.4)	15	(0.1)	(6.6)		(9.4)
	Kidney	C64	7	(0.1)	7	(0.2)	14	(0.1)	(6.1)	(10.0)	(4.4)
	Prostate gland	C61	11	(0.2)			11	(0.1)	(4.8)	(15.7)	
	Skin	C44	3	(0.0)	7	(0.2)	10	(0.1)	(4.4)	(4.3)	(4.4)
	Bladder	C67	5	(0.1)	3	(0.1)	8	(0.1)	(3.5)	(7.1)	(1.9)
	Bronchus and lung	C34	4	(0.1)	2	(0.1)	6	(0.1)	(2.6)	(5.7)	(1.3)
	Stomach	C16	1	(0.0)	4	(0.1)	5	(0.0)	(2.2)	(1.4)	(2.5)
	Liver	C22	1	(0.0)	1	(0.1)	2	(0.0)	(0.9)	(1.4)	(0.6)
	Thyroid gland	C73	0	(0.0)	2	(0.1)	2	(0.0)	(0.9)	(0.0)	(1.3)
	*Other**		*15*	*(0.2)*	*23*	*(0.6)*	*38*	*(0.4)*	*(16.6)*	*(21.4)*	*(14.5)*
**Total**			**70**	**(1.1)**	**159**	**(4.5)**	**229**	**(2.0)**	**(100)**	**(100.0)**	**(100.0)**
Liver C73	Bladder	C67	44	(0.7)	6	(0.2)	50	(0.5)	(22.4)	(26.8)	(10.2)
	Colon-rectum	C18-C21	27	(0.4)	12	(0.3)	39	(0.4)	(17.5)	(16.5)	(20.3)
	Prostate gland	C61	35	(0.5)			35	(0.3)	(15.7)	(21.3)	
	Hematopoietic system and lymph nodes	C42, C77	25	(0.4)	9	(0.3)	34	(0.3)	(15.2)	(15.2)	(15.3)
	Breast	C50	0	(0.0)	17	(0.5)	17	(0.2)	(7.6)	(0.0)	(28.8)
	Bronchus and lung	C34	4	(0.1)	2	(0.1)	6	(0.1)	(2.7)	(2.4)	(3.4)
	Stomach	C16	1	(0.0)	3	(0.1)	4	(0.0)	(1.8)	(0.6)	(5.1)
	Uterus	C53-C55			3	(0.1)	3	(0.0)	(1.3)		(5.1)
	Kidney	C64	3	(0.0)		(0.0)	3	(0.0)	(1.3)	(1.8)	(0.0)
	Skin	C44	2	(0.0)		(0.0)	2	(0.0)	(0.9)	(1.2)	(0.0)

Second Primary Cancer (SPC) site (ICD-O-3 topography code)	Site of First Primary Cancer	ICD-O-3 topography code	M n	M (%)	F n	F (%)	Total n	Total (%) of all SPC	% of First Cancer Site § overall (%)	in M (%)	in F (%)
	Pancreas	C25	1	(0.0)	0	(0.0)	1	(0.0)	(0.4)	(0.6)	(0.0)
	Thyroid gland	C73	0	(0.0)	1	(0.0)	1	(0.0)	(0.4)	(0.0)	(1.7)
	*Other**		*22*	*(0.3)*	*6*	*(0.2)*	*28*	*(0.3)*	*(12.6)*	*(13.4)*	*(10.2)*
**Total**			**164**	**(2.5)**	**59**	**(1.7)**	**223**	**(2.0)**	**(100)**	**(100.0)**	**(100.0)**
*Other**			*679*	*(10.5)*	*479*	*(13.5)*	*1,158*	*(12.0)*	*(100)*	*(100.0)*	*(100.0)*
**Grand Total**			**6,450**	**(100)**	**3,559**	**(100)**	**10,009**	**(100)**	**(100)**	**(100.0)**	**(100.0)**

Notes: ICD-O-3 International Classification of Diseases for Oncology - version 3; * *Other* [cancer sites] includes other ICD-O-3 codes; M males; F females; § number of first primary cancer by site provided in Table 1 of manuscript.

Table S6: Cancer cases (2003-2007) and percent frequency of Second Primary Cancer (SPC) (2003-2017), by sex and age

Cancer site (ICD-O-3 topography code)	Sex	Age range	Patients with First cancer	Patients with SPC	Percent frequency of SPC overall	within 6 mo.	7-60 mo.	after 60 mo.
All cancers	M	0-49	2,825	138	4.9	0.5	1.4	3.0
		50-69	11,555	1,552	13.4	1.5	4.8	7.1
		70+	14,784	1,553	10.5	2.1	5.1	3.3
		**Total**	**29,164**	**3,243**	**11.1**	**1.7**	**4.6**	**4.8**
	F	0-49	4,757	237	5.0	0.5	1.7	2.8
		50-69	9,075	848	9.3	1.0	3.7	4.7
		70+	10,544	645	6.1	0.9	2.9	2.3
		**Total**	**24,376**	**1,730**	**7.1**	**0.9**	**2.9**	**3.3**
Breast (C50)	F	0-49	1654	82	5.0	0.2	1.9	2.8
		50-69	2862	248	8.7	0.6	3.4	4.7
		70+	2256	172	7.6	0.4	3.9	3.3
		**Total**	**6772**	**502**	**7.4**	**0.5**	**3.2**	**3.8**
Colon-Rectum (C18-C21)	M	0-49	212	18	8.5	0.5	2.8	5.2
		50-69	1,398	202	14.4	1.2	5.5	7.7
		70+	2,037	234	11.5	2.7	5.5	3.3
		**Total**	**3,647**	**454**	**12.4**	**2.0**	**5.3**	**5.1**
	F	0-49	203	12	5.9	0.5	2.0	3.4
		50-69	1,080	107	9.9	1.1	4.3	4.5
		70+	1,815	128	7.1	1.5	3.1	2.4
		**Total**	**3,098**	**247**	**8.0**	**1.3**	**3.5**	**3.2**
Lung (C34)	M	0-49	170	4	2.4	0.0	0.6	1.8
		50-69	1,976	81	4.1	0.9	1.2	2.0
		70+	2,389	90	3.8	1.2	2.0	0.6
		**Total**	**4,535**	**175**	**3.9**	**1.0**	**1.6**	**1.2**
	F	0-49	118	3	2.5	0.8	0.0	1.7
		50-69	385	9	2.3	0.5	0.8	1.0
		70+	492	11	2.2	0.8	1.2	0.2
		**Total**	**995**	**23**	**2.3**	**0.7**	**0.9**	**0.7**
Prostate (C61)	M	0-49	35	4	11.4	2.9	0.0	8.6
		50-69	1905	323	17.0	2.1	5.7	9.1
		70+	2964	432	14.6	2.0	7.2	5.5
		**Total**	**4904**	**759**	**15.5**	**2.0**	**6.5**	**6.9**
Bladder (C67)	M	0-49	195	6	3.1	0.0	0.5	2.6
		50-69	1,606	350	21.8	2.4	7.6	11.8
		70+	2,160	372	17.2	3.8	8.2	5.2
		**Total**	**3,961**	**728**	**18.4**	**3.1**	**7.6**	**7.7**
	F	0-49	72	7	9.7	2.8	2.8	4.2
		50-69	248	39	15.7	1.2	6.9	7.7
		70+	436	38	8.7	2.3	2.5	3.9
		**Total**	**756**	**84**	**11.1**	**2.0**	**4.0**	**5.2**

M males, F females; age ranges: 0-49, age under 50 years old; 50-69: between 50 and 69 years old; 70+: 70 years old and older. Percent frequency of SPC was computed for all SPC after diagnosis of FPC (overall), or separately for three follow-up ranges: within 6 months, after 6 months and within 5 years (7-60 mo.), and after 5 years of follow-up (after 60 mo.).
